# A221 EFFECT OF PROXIMITY TO A SPECIALTY TERTIARY CENTRE ON OUTCOMES IN INFLAMMATORY BOWEL DISEASE: A POPULATION-BASED RETROSPECTIVE COHORT STUDY

**DOI:** 10.1093/jcag/gwac036.221

**Published:** 2023-03-07

**Authors:** M Mikail, Q Alkhateeb, V Pope, R Khanna

**Affiliations:** 1 Division of Gastroenterology, Max Rady College of Medicine at the University of Manitoba, Winnipeg; 2 Department of Medicine; 3 Division of Gastroenterology, Schulich School of Medicine & Dentistry at Western University, London, Canada

## Abstract

**Background:**

The etiology of inflammatory bowel disease (IBD) is unknown; however, developed nations such as Canada ranking amongst the highest worldwide. With many diseases patients in urban and rural areas have different access to care and resources.

**Purpose:**

To describe the differences in outpatient healthcare utilization, use of biologic agents and complication of IBD based on proximity to a tertiary health care centre.

**Method:**

A retrospective cohort study was conducted comparing IBD patients seen in IBD clinics at affiliated with Western University in London, Canada between August 2019 – December 2019. IBD patients were compared on their use of outpatient healthcare utilization, biologic agents and IBD complications based on their proximity to a tertiary care centre (>100 km and <100 km). Patients residing >100 km from a tertiary centre were termed “rural” while <100 km from a tertiary centre were termed “urban.” Retrospective chart review occurred over a six-month period between January to June 2021.

**Result(s):**

A total of 481 were reviewed. Of those, 97 (UC, n=29; CD, n=68) and 95 (UC, n=30; CD, n=65) met inclusion for the urban and rural groups respectively. Patient demographics were similar between the two groups except IBD disease location with pancolitis seen more commonly in urban patients compared to ileocolonic in rural patients (urban, n=39; rural, n=34). IBD patients in both groups had similar number of appointments (urban, n=20.1 ± 13.8; rural, n=17.5 ± 12.1) and endoscopic procedures (urban, n= 4.9 ± 3.1; rural, n= 4.7 ± 3.2) with their gastroenterologists. More urban patients were managed with no therapy for their IBD (urban, n=16; rural, n=5). A higher rate of rural patients were managed with biologics (urban, n=56; rural, n=66) and combination therapy (urban, n=16; rural, n=27). The most common related IBD-related complications were IBD flares (urban, n=55; rural, n=60), intestinal strictures (urban, n=25; rural, n=34), intestinal obstructions (urban, n=10; rural, n=23) and rectal/genitourinary fistulas (urban, n=6; rural, n=21). Similar numbers of intra-abdominal surgery were seen between both groups with partial bowel resection (urban, n=13; rural, n=12) and right hemicolectomy (urban, n=10; rural, n=18) as the predominant surgery in urban and rural patients, respectively.

**Conclusion(s):**

This study demonstrated outpatient healthcare utilization when attending specialty gastroenterology appointments and outpatient endoscopies were numerically similar in rural and urban patients. IBD patient residing further from a tertiary care centre were numerically more likely to be managed with biologics and combination therapy. However the dataset is small and generalizations cannot be made.

**Please acknowledge all funding agencies by checking the applicable boxes below:**

None

**Disclosure of Interest:**

None Declared

